# Exploring community-level gut health amidst COVID-19 pandemic: a proposed application of longitudinal wastewater surveillance

**DOI:** 10.1007/s11356-026-38012-3

**Published:** 2026-08-01

**Authors:** Emma Lancaster, Yuehan Ai, Jiyoung Lee

**Affiliations:** 1 Division of Environmental Health Sciences, College of Public Health, The Ohio State University, Columbus, OH USA; 2 Environmental Sciences Graduate Program, The Ohio State University, Columbus, OH USA; 3 Department of Food Science & Technology, The Ohio State University, Columbus, OH USA; 4 Infectious Diseases Institute, The Ohio State University, Columbus, OH USA

**Keywords:** Environmental monitoring, Water microbiology, Human gut microbiota, Epidemiology, SARS-CoV-2, Environmental health

## Abstract

Wastewater-based epidemiology (WBE) offers a unique, scalable method to monitor disease burden at a community level by capturing all individuals within a sewershed. While emerging evidence suggests that SARS-CoV-2 may influence the human gut microbiota, which is essential for health and disease outcomes, most microbiome studies remain limited to the individual scale. Given wastewater’s sensitivity to fluctuations in human fecal composition, we propose leveraging WBE as a cost-effective tool to characterize longitudinal microbial shifts in community-level gut microbiota throughout the COVID-19 pandemic. In this exploratory study, we analyze wastewater from two central Ohio, USA, cities, Columbus and Newark, which differ in socioeconomic and demographic characteristics. Monthly influent samples (*n* = 69) were collected from August 2020 through June 2022. Clinical COVID-19 cases were collected from within each sewershed, and SARS-CoV-2 gene concentrations were quantified from wastewater samples. 16S rRNA gene sequencing was conducted to characterize human-gut–associated bacterial communities. We report that wastewater influent exhibits distinct genus-level bacterial signatures reflective of each catchment population. Significant temporal changes in bacterial structure and diversity were observed across both cities, indicating community gut health shifts throughout the pandemic. Several taxa, including but not limited to *Collinsella*, *Megasphaera*, and *Actinobacteriota*, showed notable relative abundance fluctuations that may be linked to infection and warrant further investigation as potential microbial biomarkers. This study demonstrates that urban population-level gut microbiome patterns can be robustly characterized through wastewater influent. Most notably, this 23-month study represents the first effort to examine community gut microbiota structural changes across two cities during the COVID-19 pandemic. Our findings highlight sewage as a population-level proxy for public health status and disease burden while offering a novel framework for integrating microbiome science into WBE. This study underscores the potential of wastewater surveillance to advance global infectious disease preparedness and population-scale microbiome research.

**Graphical Abstract:**

**Figure:**
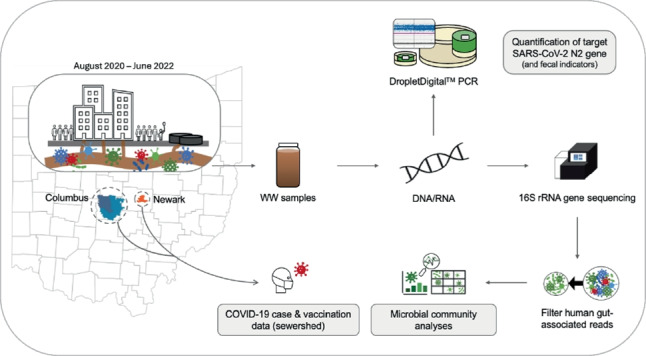

**Supplementary Information:**

The online version contains supplementary material available at 10.1007/s11356-026-38012-3.

## Introduction

The gastrointestinal (GI) tract is the largest reservoir of human microbes, housing an estimated 70–80% of the body’s immune cells, and plays a critical role in systemic immune regulation and inflammation (Donaldson et al. 2016; Wiertsema et al. 2021). Human gut microbiota varies widely across individuals and is shaped by many sociodemographic factors including lifestyle, medications, diet, ethnicity, age, and geography (Hall et al. 2017; Shanahan et al. 2021). Increasingly recognized as a key indicator of health, gut microbiome (GM) imbalances (dysbiosis) contribute to diseases such as diabetes, autoimmune disorders, inflammatory bowel disease, chronic obstructive pulmonary disease, mental illnesses, and cardiovascular conditions (de Goffau et al. 2013; Enaud et al. 2020; Jamshidi et al. 2019; Kleiman et al. 2015; Wang et al. 2011). Through the gut-lung axis, gut-derived short-chain fatty acids and other metabolites modulate respiratory immune responses and influence susceptibility, severity, and recovery from viral infections, including COVID-19. Clinical studies have shown that gut dysbiosis is associated with heightened respiratory inflammatory responses and altered microbial gut composition in patients with respiratory infections, further underscoring the importance of understanding GM dynamics during the COVID-19 pandemic (Brown et al. 2017; Budden et al. 2017; Dang and Marsland 2019; Dhar and Mohanty 2020; Zhang et al. 2020).

Given this gut-lung axis, the GM is hypothesized to influence COVID-19 immune responses. COVID-19 persisted as a global pandemic from 2020 to 2023, with over 778 million cases worldwide and 103 million in the USA as of July 2025 (Centers for Disease Control and Prevention (CDC) 2023; United Nations (UN) 2023; World Health Organization (WHO) 2025). Since SARS-CoV-2 is shed via feces and respiratory secretions, wastewater-based epidemiology (WBE) rapidly emerged as a scalable, real-time surveillance method to monitor infection (Hill et al. 2020; McClary-Gutierrez et al. 2021). Compared to traditional clinical reporting, WBE provides a non-invasive, cost-effective approach that captures symptomatic, asymptomatic, and presymptomatic infections while minimizing reporting biases (Sims and Kasprzyk-Hordern 2020). Despite wastewater’s complex, microbe-heavy matrix, microbial signals remain highly sensitive, capable of detecting the minor fluctuations in microbial abundances (Singh et al. 2024). In response, the Ohio Department of Health (ODH) and the Ohio Environmental Protection Agency (OEPA) launched the Ohio Coronavirus Wastewater Monitoring Network (OCWMN) in July 2020 to track SARS-CoV-2 trends and inform local interventions (Bohrerova et al. 2023; Davis et al. 2023).

Clinical studies report decreased gut microbial diversity and persistent dysbiosis in COVID-19 patients compared to healthy individuals, even following post-recovery (Chakraborty et al. 2022; Farsi et al. 2022; Gou et al. 2021; Hazan et al. 2022; Yeoh et al. 2021; Zuo et al. 2020a). COVID-19 severity is further influenced by comorbidities including obesity, diabetes, and hypertension, which are associated with chronic inflammation and gut dysbiosis (Dhar and Mohanty 2020; Donati Zeppa et al. 2020; Gou et al. 2021; Zhang et al. 2023). Therefore, gut microbiota likely influences COVID-19 virulence, demonstrating that SARS-CoV-2 may also play a critical role in GM dysbiosis (Farsi et al. 2022). However, most GM studies rely on small cohorts or animal models due to cost and feasibility, limiting generalizability (Vandeputte et al. 2017). Large-scale studies are needed to capture microbial diversity across demographic and geographical groups (Bowyer et al. 2019; Kwak et al. 2024; Miller et al. 2016) and to identify robust associations between the GM and chronic conditions (Debelius et al. 2016). Longitudinal cohort designs are particularly needed to assess the temporal stability of gut microbiota, yet the financial and logistical demands often present other challenges. Population-level microbiome research may advance to reveal early biomarkers for infectious and chronic diseases and inform tailored prevention strategies, while environmental and lifestyle factors (e.g., sanitation, pollution, urbanization, diet, and smoking) continue to shape microbial communities (Panthee et al. 2022). Given the limitations of traditions GM cohorts, WBE presents an underexplored and scalable, cost-effective alternative for monitoring community-level GM health.

In this pilot study, we address critical gaps in population-scale microbiome research and public health surveillance by presenting a novel and timely investigation into leveraging WBE to explore community-level gut microbiota shifts during the COVID-19 pandemic. Specifically, we evaluate whether wastewater-derived GM profiles capture community-level biological patterns during the pandemic and examine their potential relevance for population health surveillance. Our first objective is to characterize the collective GM of populations in community wastewater from two socio-spatially different cities within central Ohio (OH) over time. Since SARS-CoV-2 can be shed in feces and alter the gut microbiota at the individual level, we assess whether local surges of COVID-19 infection patterns may be observed in wastewater-derived community gut microbial composition. Therefore, our second objective is to analyze how community gut microbiota shifted throughout the COVID-19 pandemic across both Ohio cities. Our 23-month sampling period (August 2020–June 2022) across three wastewater treatment plants (WWTPs) in two Ohio cities (Columbus and Newark) provides rare temporal and geographic resolution, enabling robust analysis of microbiota dynamics across COVID-19 pandemic phases. This exploratory design is particularly valuable given the scarcity of long-term, cross-community microbiome studies during COVID-19. By integrating 16S rRNA gene sequencing, droplet digital PCR for SARS-CoV-2 N2 gene quantification, and clinical COVID-19 case data, we establish a holistic framework linking microbial shifts with population disease burden and demonstrate the broader potential of WBE for monitoring collective community gut health during disease outbreaks. Ultimately, these findings enhance our understanding of how community health and infectious disease dynamics interact and provide a foundation for future large-scale wastewater microbiome surveillance efforts.

## Materials and methods

### Study selection and wastewater sampling

**Fig. 1:**
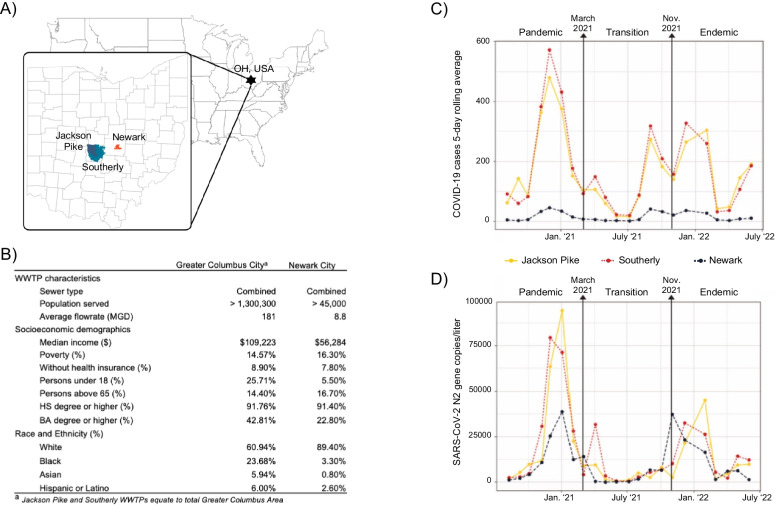
Background information on study area located in central Ohio, USA. **A** Three WWTP sewershed boundaries (Jackson Pike: dark blue; Southerly: light blue; Newark: red) overlapping Ohio county boundaries outlined in gray. **B** WWTP characteristics and city demographics. Longitudinal timeline of **C** COVID-19 cases (5-day rolling average) and **D** SARS-CoV-2 N2 (gene copies per liter) wastewater concentrations from August 2020 to June 2022

To evaluate potential differences in community gut microbiome using wastewater, we selected two cities based on the following criteria: (i) sampling locations represent sociodemographic distinguishable communities, (ii) all wastewater treatment plants (WWTPs) operate using the same sewer system type, and (iii) the cities differ in COVID-19 disease burden. These criteria result in three catchment areas in two spatially distinct central Ohio cities, Columbus and Newark (Fig. 1). Two WWTPs serving Columbus (Jackson Pike and Southerly) serve the capital city of Ohio, Columbus, with a catchment area population of greater than 1.3 million, whereas the Newark facility serves a population of approximately 45,000 (Fig. 1B). The cities also differ in sociodemographic characteristics, with Columbus having a median household income of $109,223 compared to Newark’s $56,284, alongside addition variances in poverty, education, and racial demographics (Fig. 1B). All three WWTPs use a combined sewer system, ensuring comparable transport of sanitary domestic sewage and stormwater through a single-pipe system. As shown in Fig. 1C, D, these two cities have variations in confirmed COVID-19 case rates and SARS-CoV-2 gene concentrations via wastewater influent (Fig. 1C, D).

The sampling period began in early August 2020 and ended the first week of June 2022. One liter 24-h composite samples of untreated wastewater influent were collected from each WWTP approximately during the first week of each month. Monthly sampling was selected to capture long-term, population-level trends in community gut microbiota across pandemic phases. SARS-CoV-2 viral immunity effectiveness is negligent in the first 2 weeks, reaching peak protectiveness in infection after 3 weeks (Chemaitelly et al. 2021). Therefore, since SARS-CoV-2 immune response develops over multi-week timescales and community-level infection dynamics shift gradually, monthly sampling provides an appropriate temporal resolution for assessing broad microbial changes rather than short-term fluctuations. This design aligns with prior longitudinal WBE studies and reflects our focus on pandemic temporal-scale patterns rather than daily variability (Cluzel et al. 2026; Tharak et al. 2022).

**Fig. 2:**
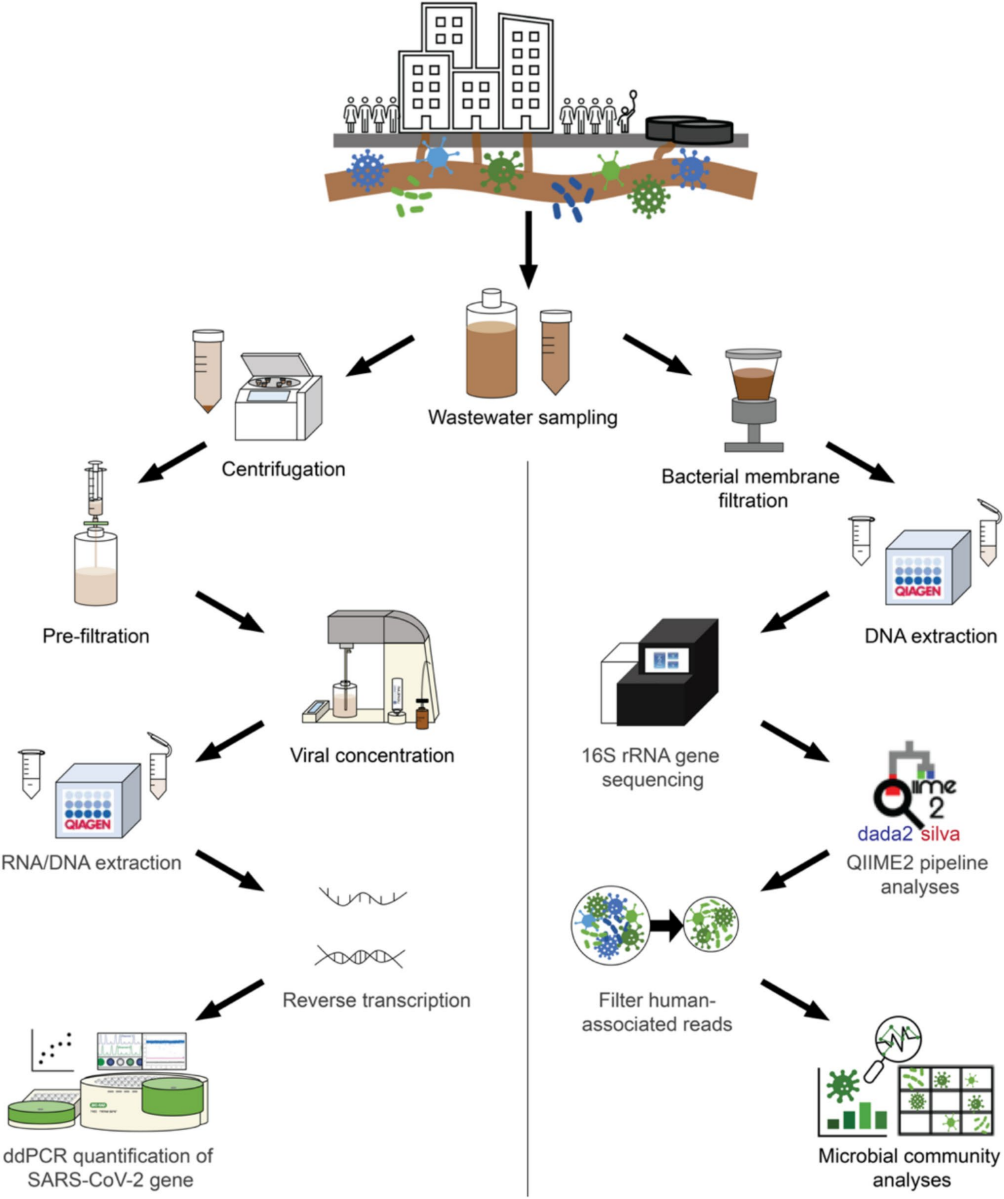
Schematic representation of study methodology, including sample collection and processing. Image created using Microsoft PowerPoint (v. 16.82) and Adobe Illustrator (v. 28.2)

All samples were delivered to the Environmental Microbiology and One Health Lab (EMOHL), College of Public Health, the Ohio State University and processed on the day of sampling. Schematic representation of subsequent sample processing methodology is shown in Fig. 2.

### Wastewater processing: concentrating viruses and bacterial communities

Undiluted 50 mL aliquots of wastewater influent samples were filtered through sterile bacterial 0.2 µm polycarbonate membrane filters (Millipore, Burlington, MA, USA) in duplicate. In order to concentrate the SARS-CoV-2 virus from wastewater influent samples, each sample was processed in duplicate following a solid removal and viral concentration protocol. First, for solid removal and detaching viral particles from solid, 0.05% Tween-20 was added to 100 mL of undiluted wastewater samples and centrifuged at 4 °C, 2500 × g for 10 min. Afterwards, the supernatant underwent pre-filtration using a 0.45 µm sterile filter unit (Millipore, Burlington, MA, USA) to remove small debris and bacteria. The filtrate was then concentrated using a 0.05-µm Hollow Fiber Polysulfone Concentrating Pipette Select tips (Innovaprep, Drexel, MO, USA). Two additional negative controls of sterile PCR-grade water (50 mL) were concentrated in duplicate. Following the manufacturer’s instructions, including valve open 600 ms, 1 pulse, 10 foam factor, valve start time 3.0 s, 25% pump, delay time of pump 1 s, approximately 200–250 µL of viral concentrate was eluted. The viral eluent was then used for RNA/DNA extraction or stored at − 80 °C for further analyses. All wastewater samples were processed on the day of sampling following the methods of Ai et al. 2021, where more detailed laboratory procedure and viral recovery efficiency for this method can be found.

### RNA/DNA extraction and droplet digital PCR

For wastewater microbiome analyses, microbial DNA was extracted from the bacterial 0.2 µm membrane filters using the DNEasy PowerSoil Pro Kit (Qiagen, Hilden, Germany). The final microbial DNA eluent produced 100 µL per sample. DNA concentration and quality were measured using the NanoDrop™ 2000 Spectrophotometer (Thermo Fisher) prior to 16S rRNA gene sequencing.

For SARS-CoV-2 quantification, nucleic acid was extracted from the viral eluent concentrate using the RNEasy PowerMicrobiome Kit, along with the two negative controls (Qiagen, Germantown, MD, USA). Next, the nucleic acid underwent reverse transcription with the High-Capacity cDNA Reverse Transcription Kit (Applied Biosystems, Pittsburgh, PA, USA). Afterwards, 3.0 µL of cDNA was used for the quantification of the SARS-CoV-2 genome using a droplet digital PCR (ddPCR) system (Bio-Rad system). The ddPCR assay targeted the nucleocapsid N2 gene region of SAR-CoV-2 using primers/probe sets from the US Centers for Diseases Control and Prevention (CDC) (Hirotsu et al. 2020; Jung et al. 2020). To account for temporal fluctuations in wastewater fecal load and to ensure that exploratory biomarkers were not simply reflecting fecal pollution, we quantified two common human viral fecal indicators pepper mild mottle virus (PMMoV) and cross-assembly phage (crAssphage), using diluted cDNA. Because recent studies report conflicting findings of normalizing wastewater signals, and our previous work demonstrated that normalization to these indicators was unnecessary at our sampling locations, we used PMMoV and crAssphage only to verify that intrinsic marker abundances were not driven by variation in human fecal loads (Ai et al. 2021; Schmiege et al. 2024). Details regarding assay reaction mixture, system control, and PCR inhibition can be found in Ai et al. (2021). The QX200 droplet generator was used to generate droplets prior to target gene amplification using the Bio-Rad C1000 Touch Thermal Cycler with a denaturing step for 10 min at 94 °C, followed by 40—45 cycles for 30 s and annealing at 94 °C, a closing incubation for 10 min at 98 °C, and a final 4 °C hold. Subsequently, for each ddPCR assay, a QX200 droplet reader (V 1.7, Bio-Rad) was used for gene concentration quantification in duplicate and two technical replicates. For each assay, the limit of detection (LOD) is one gene copy/reaction, and the limit of quantification (LOQ) is two gene copies/reaction (LOQ). Target gene copy numbers were calculated as the average of the two biological and two technical samples.

### 16S rRNA gene sequencing and microbial community analyses

The sixty-nine samples from wastewater influent of the three WWTPs in central Ohio (Fig. 1) underwent paired-end 16S rRNA gene sequencing. The V4–V5 region of the 16S rRNA gene was sequenced at the Molecular and Cellular Imaging Center at the Ohio State University (Wooster, OH) on the Illumina MiSeq platform using the primers 515 F (5′-GAGTGCCAGCMGCCGCGGTAA-3′) and 806R (5′-ACGGACTACHVGGGTWTCTAAT-3′). Raw reads were processed and filtered with quality-trimmed (cutoff at 240 base pair (bp) for forward and reverse) FASTQ files in QIIME2 using DADA2 for amplicon sequence (ASV) determination and deposited in the National Center for Biotechnology Information (NCBI) Sequence Read Archive (SRA) under the accession number PRJNA1405769 (Bolyen et al. 2019; Callahan et al. 2016). To normalize for diversity analyses, data were rarefied and sampled at a depth of 7190 to retain all samples, and taxonomy was classified using the 99% SILVA database (Glöckner et al. 2017). Subsequently, taxa were filtered using the Su et al. (2017) database to retain human gut-associated reads (defined as the bacterial taxa detected in the human intestinal tract) and omit wastewater-associated reads which are known to undergo seasonal variation (LaMartina et al. 2021), in order to characterize the human GM in wastewater influent. Notably, this database includes 382 bacterial-specific genera from the Metagenomics of the Human Intestinal Tract (MetaHit) (European Commission 2019) project and the Human Microbiome Project (HMP) (NIH Human Microbiome Project (HMP) 2024), encompassing nearly all genera of human gut bacteria with a 16S rRNA gene catalogue reference set. Sequencing depth of all reads, human gut-associated filtered reads, and proportion of human gut-associated reads can be found in Supplementary Table 1. Afterwards, the following alpha diversity metrics, Shannon’s Index, Faith’s Phylogenetic Diversity, Observed Features, and Pielou’s Evenness, were calculated using the q2-diversity plugin via QIIME2 (Bolyen et al. 2019). Kruskal–Wallis tests were used to determine global significance (*p* < 0.05), and additional Wilcoxon rank sum tests with Bonferroni adjusted *p*-value were used to evaluate pairwise significant differences (*p* < 0.05). Beta diversity metrics, Bray–Curtis distance, Jaccard distance, and weighted and unweighted UniFrac distances were assessed using the same q2-diversity plugin with PERMANOVA tests to evaluate significance (*p* < 0.05). We used two statistical tools, linear discriminant analysis effect size (LEfSe) (Paulson et al. 2013) (https://huttenhower.sph.harvard.edu/galaxy) and analysis of differential abundance (ALDEx2) (https://www.bioconductor.org/packages/devel/bioc/vignettes/ALDEx2/inst/doc/ALDEx2_vignette.html), for identifying differentially abundant microbes at the phylum, family, and genus level to ensure confidence of results. LEfSe uses non-parametric factorial Kruskal–Wallis tests between groups, while ALDEx2 uses a more robust Monte Carlo approach with different normalization instances.

### Additional data collection, preparation, and statistical analyses

Clinical COVID-19 cases were recovered from the Ohio Department of Health (ODH) COVID-19 Dashboard which houses the most recent new and cumulative cases from a wide array of partner agencies including, but not limited to, Developmental Disabilities, Veteran’s Homes, Youth Services, Mental Health and Addiction Services, and Rehabilitation and Corrections (ODH 2022). Cases reflect the confirmed case counts by the estimated symptom onset date within the boundary of each WWTP catchment. Our previous study (Ai et al. 2021) concluded that when comparing 3-day, 5-day, and 7-day rolling average of case counts, the 5-day rolling average reflected the highest Spearman correlation rank coefficient with respective SARS-CoV-2 N2 gene wastewater concentrations. Therefore, we used the 5-day rolling average of confirmed clinical COVID-19 cases in this study.

To enable temporal analysis across the study period, we stratified sampling dates into defined pandemic categories. Grouping samples into these pandemic phases was necessary to conduct statistical comparisons of microbial (alpha and beta) diversity and relative and discriminant abundances over time. As this work has never been done before, we decided to stratify pandemic groupings using SARS-CoV-2 vaccination rates. We selected vaccination rate-based stratification because rising vaccination coverage reflects fundamental shifts in community-level immunity, which influences transmission dynamics, infection burden, and public health interventions. Prior epidemiological studies have shown that higher vaccination coverage correlates with reductions in SARS-CoV-2 transmission and reduced incidence at the population level (Au 2022; McLaughlin et al. 2022). In this context, using vaccine rates as a stratification metric anchors our microbial community comparisons to significant shifts in population immunity and disease burden, rather than arbitrary calendar dates. We also took into consideration the statewide and county-level health responds in comparison to pandemic phases and vaccination rates. Thus, our stratification decision is biologically and epidemiologically justified, given the limited preliminary work and data. The resulting categorical variable based on vaccination-driven phases was used in subsequent statistical analyses of WW microbial community composition. Supplementary Fig. 1 provides additional context, including a visualization of the defined pandemic phases, county-level vaccination proportions, and key governmental public health interventions spanning the sampling period (August 2020–June 2022).

Vaccination data was collected from the ODH COVID-19 Dashboard (ODH 2022). Jackson Pike and Southerly WWTPs are located in Franklin County, and Newark WWTP is located in Licking County. Both counties began reporting vaccination rates on 12/14/2020. For each county, we calculated the county population vaccinated from 12/14/2020 through 06/01/2022. We then aligned vaccinate data with the WWTP sampling dates and examined the cumulative vaccination curves to identify major shifts in the linear slope. These inflection points were used to define three pandemic phase categories: pandemic (08/09/2020–03/02/2021), transition (04/04/2021–10/03/2021), and endemic (11/02/2021–06/01/2022). The sample distribution (*n* = 69 total) across groups was balanced, with 24 samples in the pandemic phase (8 sampling dates), 21 in the transition phase (7 sampling dates), and 24 in the endemic phase (8 sampling dates). Additionally, vaccination trends further supported stratification, as Franklin and Licking counties surpassed 50% cumulative vaccination during the shift from transition to endemic phase, marking a clear epidemiologic phase change. In Franklin County, vaccination levels remained minimal during the pandemic phase (slope < 0.001), increased sharply during the transition phase (slope > 0.01), and plateaued during the endemic phase (slope < 0.0001). This initial analysis of vaccination rate was conducted to establish the pandemic phase variable for subsequent statistical microbial analyses.

As previously noted, clinical studies have denoted significant shifts in the relative abundances of specific microbiota during infection. Building on these findings, we sought to further explore whether similar patterns could be observed at the population level. To do this, we conducted correlation analyses to examine potential bacterial biomarkers associated with SARS-CoV-2 infection. Taxa that were differentially abundant taxa across pandemic phase were analyzed in correlation to SARS-CoV-2 N2 gene wastewater concentrations, human fecal viral indicator wastewater concentrations (PMMoV, crAssphage), reported (5-day rolling average) COVID-19 cases, and microbial alpha diversity using non-parametric Spearman correlation tests (*p* < 0.05). A correlation heat matrix was generated to visualize associations between key biomarker relative abundances, microbiome diversity indices, COVID-19 cases, and SARS-CoV-2 N2 gene wastewater concentrations. All statistical and correlation analyses were conducted in R (v.2023.12.1 + 402).

## Results

### SARS-CoV-2 gene concentrations and COVID-19 cases in central Ohio

During the 23-month study, the SARS-CoV-2 N2 gene was detected in 68/69 samples, with the one zero detection being reported from Newark WWTP on 05/02/2021. Wastewater SARS-CoV-2 N2 gene concentrations ranged from 1.91 × 10^2^ to 3.15 × 10^5^ gene copies/L of wastewater, without the non-detect exception of Newark (Fig. 1D). Wastewater concentrations follow an overall similar trend across all three WWTPs during the study phase, observing two large peaks in viral wastewater concentrations during the December/January winter sampling dates of both 2021 and 2022. Following the introduction of vaccinations to the sewershed populations, wastewater concentrations substantially dropped following early 2021 and plateaued throughout the transition phase (March 2021–November 2021). Subsequently, concentrations begin to slowly increase into the endemic phase with county cumulative vaccinations completion > 50%, but concentration levels never reached as high as the first peak prior to vaccinations introduced to the populations.

Meanwhile, confirmed COVID-19 5-day rolling averages ranged from 1.6 to 1800.4 cases per sampling date within all three WWTP sewershed boundaries. The overall case trend of Newark remained relatively stable throughout the study period, while the COVID-19 5-day rolling average within the WWTPs of Columbus, Jackson Pike and Southerly, observed no steady pattern. The Columbus WWTPs witnessed three sharp peaks in cases during the following sampling periods: (1) December/January 2020, (2) September 2021, and (3) December/January 2022. However, it is apparent that SARS-CoV-2 wastewater concentrations and COVID-19 case rolling averages follow similar trends throughout the study period. Spearman correlation analyses confirm a strong association between SARS-CoV-2 (gene copies/liter) N2 gene wastewater concentrations and clinical COVID-19 cases (5-day rolling average) within each WWTP location (*p* < 0.001) (Supplementary Fig. 2). Similar to the SARS-CoV-2 N2 gene, the two human fecal indicators, PMMoV and crAssphage, were quantified across the study period using wastewater influent to distinguish identified potential biomarkers from common indicators of fecal pollution. PMMoV was detected in all samples, with wastewater concentrations ranging from 3.83 × 10^4^ to 4.35 × 10^6^ gene copies/L of wastewater. CrAssphage was detected at even higher levels across all samples, with wastewater concentrations ranging from 6.01 × 10^5^ to 1.29 × 10^8^ gene copies/L of wastewater.

### Microbial community differences by location

**Fig. 3:**
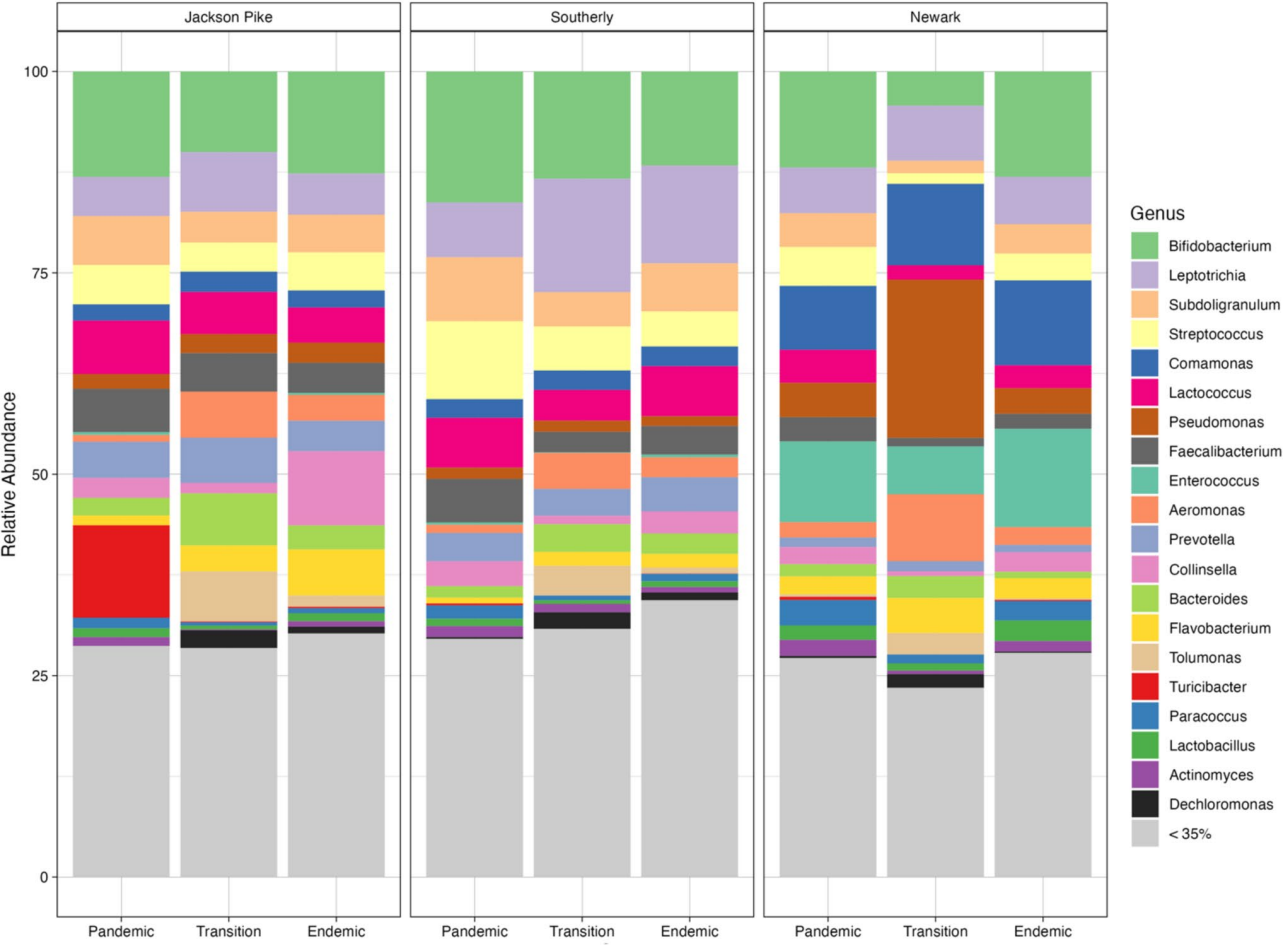
Microbial community composition. Relative abundances (%) of bacterial taxa at the genus level by WWTP and pandemic phase

Average sequencing depth of all 69 samples was 16,174 reads (Supplementary Table 1). Upon using the Su et al. (2017) database to filter for human gut-associated reads against wastewater-associated reads, an average of 6172 (38% of all total reads) human gut-associated reads was attained. Eleven unique phyla were identified for all samples, with the top four dominant phyla, Proteobacteria, Firmicutes, Actinobacteriota, and Bacteroidota, composing between 87.63 and 92.96% of the total microbial community (Supplementary Fig. 3A). There is broad consensus in the clinical microbiome field that these four phyla are the primary members of the human gut (Khanna and Tosh 2014). However, this study reports taxonomic differences emerge at the genus level by WWTP (Fig. 3) and city (Supplementary Fig. 3B). A total of 106 unique genera were retained across all samples following filtration against the Su et al. (2017) database including 382 unique genera. The top two contributing genera by WWTP were *Bifidobacterium* and *Leptotrichia*, but the similarities end there with the descending genera varying in relative abundances (Fig. 3). Compared to WWTP, genus variation was more apparent by city within the top 20 genera (Supplementary Fig. 3B). Although Shannon Index, a measure of alpha diversity, was not significant by city, the greater city of Columbus had a relatively higher Shannon Index mean than Newark city (Supplementary Fig. 4A). Likewise, the other three alpha diversity indices, Pielou’s Evenness, Faith’s Phylogenetic Diversity, and Observed Features, were relatively higher for Columbus city compared to Newark city but not statistically different at the city level (Wilcoxon rank sum *p*-value > 0.05). Similarly, all alpha diversities were not significant by WWTP, but the Jackson Pike and Southerly WWTPs had slightly higher values and ranges compared to Newark WWTP. Meanwhile, two beta diversity indices, weighted and unweighted UniFrac distances, were both significant (PERMANOVA, *p* < 0.001) by WWTP (Supplementary Fig. 4B) and city group. Differentially abundant taxa by city and WWTP were also identified at the phyla and genus level (Supplementary Table 2).

### Longitudinal microbial shifts throughout the pandemic

**Fig. 4:**
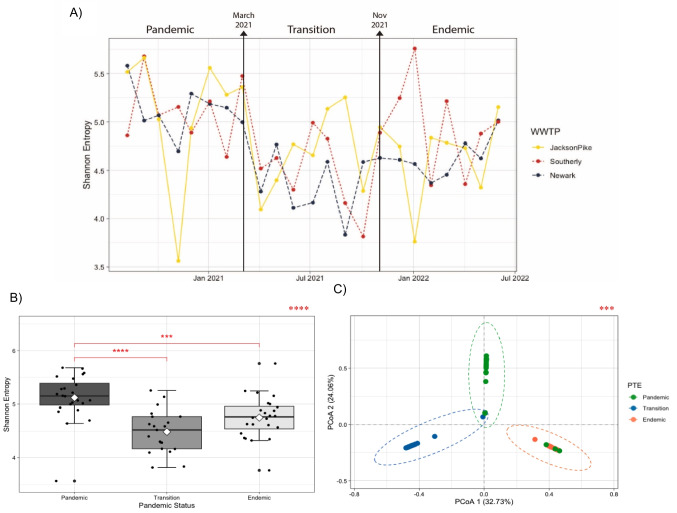
Microbial community diversity. **A** Alpha diversity (Shannon’s Index) longitudinally by WWTP location. **B** Alpha diversity (Shannon’s Index) by pandemic phase. White diamond represents average group value; global Kruskal–Wallis value is shown by comparing all groups; individual Wilcoxon pairwise comparisons are shown via asterisks. **C** Beta diversity (Bray–Curtis) by pandemic phase; PERMANOVA test value is shown via asterisks. Significance levels indicated * < 0.05, ** < 0.01, *** < 0.001, and **** < 0.0001

Distinct differences in microbial composition were found at the genus level when analyzing by pandemic phase over time (Fig. 3). Alpha diversity (Shannon’s Index) drastically varied by WWTP longitudinally from August 2020 through June 2022 (Fig. 4A). Shannon’s Index was significant by pandemic phase (Kruskal–Wallis, *p*-value < 0.0001), with the pandemic phase having a significantly higher alpha diversity index compared to both the transition (Wilcoxon, *p*-value < 0.0001) and endemic phases (Wilcoxon, *p*-value < 0.001) (Fig. 4B). For the beta diversity of bacterial communities, the Bray–Curtis dissimilarity metric reveals distinct clustering between the pandemic groups (Fig. 4C), and PERMANOVA test also reports a significant difference between communities (*p*-value < 0.001). Seasonal variation showed minor effects on beta diversity, which were reportedly weaker than those associated with pandemic stratification (Supplementary Fig. 5).

**Fig. 5:**
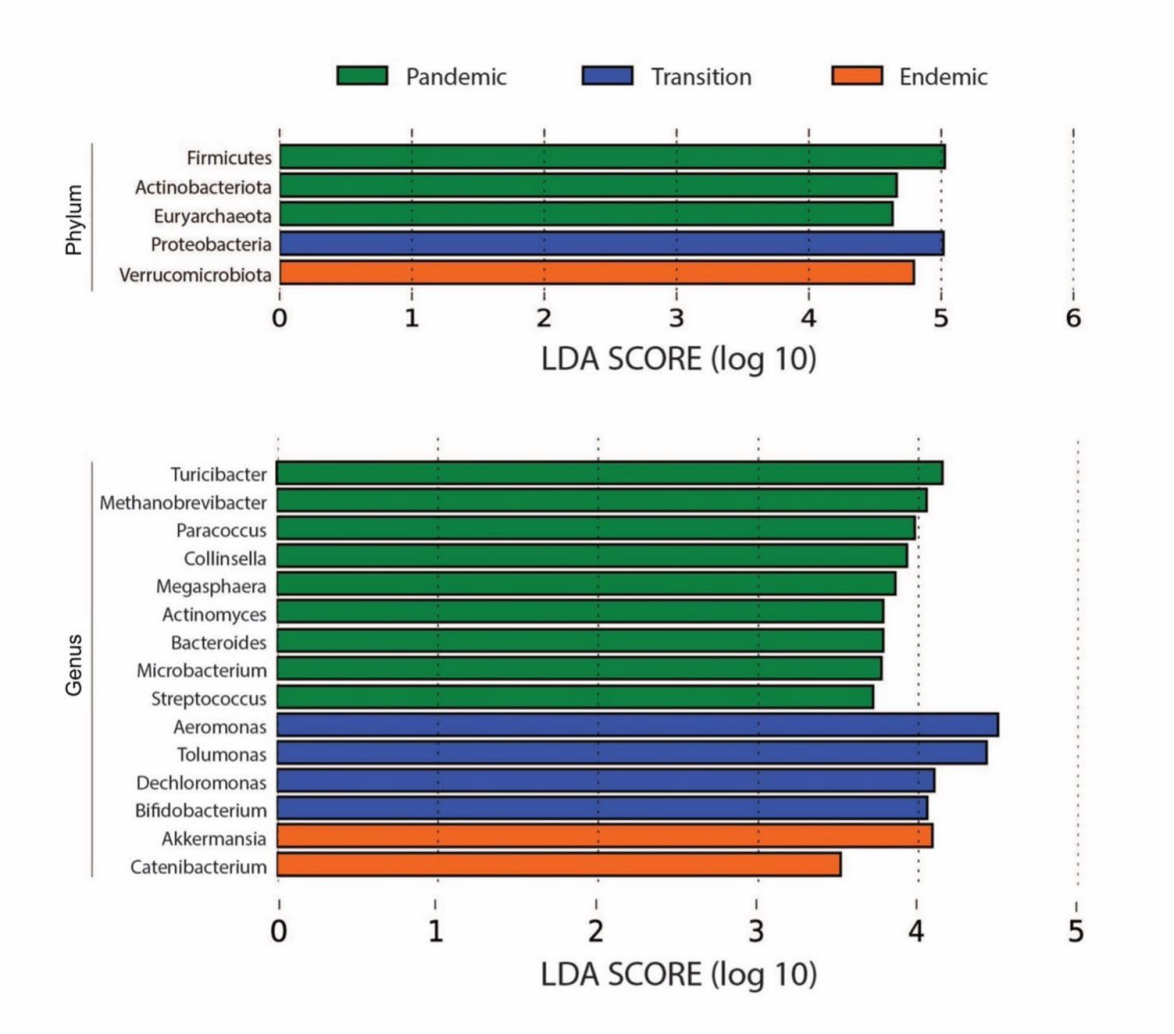
Bacterial phyla and genera with differential abundances by pandemic phase identified with LEfSe and ALDEx2. Phyla and genera in green were significantly enriched during the pandemic period, phyla and genera in blue were significantly enriched during the transition period, and phyla and genera in red were significantly enriched during the endemic period

Differences by pandemic phase revealed many differentially abundant taxa, identifying five phyla and 15 genera (Fig. 5). Firmicutes, Actinobacteriota, and Euryarchaeota were relatively more abundant during the pandemic phase. Proteobacteria were relatively more abundant in the transition phase, while Verrucomicrobiota were relatively more abundant in the endemic phase. Meanwhile, 9 of the 15 unique genera identified were relatively more increased during the pandemic phase, including in descending significance order, *Turicibacter*, *Methanobrevibacter*, *Paracoccus*, *Collinsella*, *Megasphaera*, *Actinomyces*, *Bacteroides*, *Microbacterium*, and *Streptococcus*. Three genera, *Aeromonas*, *Tolumonas*, and *Dechloromonas*, were relatively more abundant during the transition phase, and two genera, *Akkermansia* and *Catenibacterium*, were relatively more abundant during the endemic phase. Across all samples, Firmicutes, Proteobacteria, and *Aeromonas* showed the three highest log_10_ transformed LDA scores, indicating the largest distinction in taxon relative abundance within the respective group comparison.

### Microbial associations with measures of disease

**Fig. 6:**
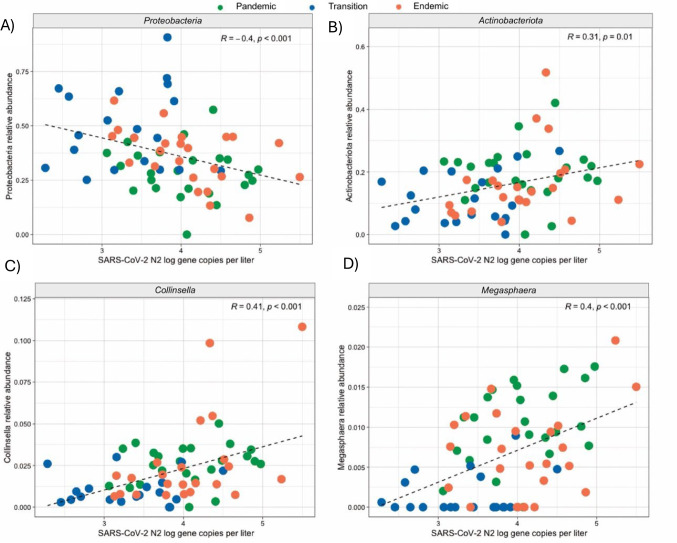
Key bacterial biomarker correlation with SARS-CoV-2 (gene copies/liter) wastewater concentrations. There were significant correlations between the relative abundance of two phyla, **A** Proteobacteria and **B** Actinobacteriota, and two genera, **C** *Collinsella* and **D** *Megasphaera* with SARS-CoV-2 N2 concentrations (Spearman correlation, *p* ≤ 0.01). The relative abundance of these four taxa (1) do not correlate with fecal indicator viruses (crAssphage and PMMoV) wastewater concentrations (Spearman correlation, *p* > 0.05) and (2) are significant by pandemic phase (differential abundance testing LEfSe and ALDEx2; Kruskal–Wallis *p* ≤ 0.01)

The relative abundance of the 20 identified differentially abundant taxa by pandemic phase was further analyzed to assess their potential as wastewater-based bacterial biomarkers of SARS-CoV-2 infection. Since wastewater is highly variable (i.e., flow rate, dilution, and rainfall), these unique taxa underwent preliminary analyses to confirm zero correlation (Spearman correlation *p*-value > 0.05) with fecal indicators (crAssphage and PMMoV log gene copies/L) wastewater concentrations. This step ensured that taxa abundance was independent of fecal pollution levels and, therefore, not just a proxy for overall fecal concentration. Afterwards, these taxa were then evaluated to determine whether their abundances were differentially significant by pandemic phase (differential abundance testing LEfSe and ALDEx2; Kruskal–Wallis *p* ≤ 0.01). Of the taxa that fulfilled the previous criteria, final Spearman correlation analyses reveal that the relative abundance of two phyla and two genera is significantly associated (*p*-value ≤ 0.01) with SARS-CoV-2 N2 wastewater concentrations (Fig. 6). Proteobacteria negatively correlate with SARS-CoV-2 levels (*p* < 0.001), and SARS-CoV-2 levels positively correlate with the other three genera, *Actinobacteriota* (*p* ≤ 0.01), *Collinsella* (*p* ≤ 0.001), and *Megasphaera* (*p* ≤ 0.001). Notably, the varying relative abundances of these four key taxa appear to cluster by pandemic phase with respect to SARS-CoV-2 wastewater concentrations (Fig. 6), further supporting the LEfSe and ALDEx2 differential abundance conclusions. While it is essential for these taxa to undergo validation with more extensive wastewater samples outside of this study, these two phyla and two genera may serve as useful biomarkers for strengthening SARS-CoV-2 surveillance and early outbreak detection.

**Fig. 7:**
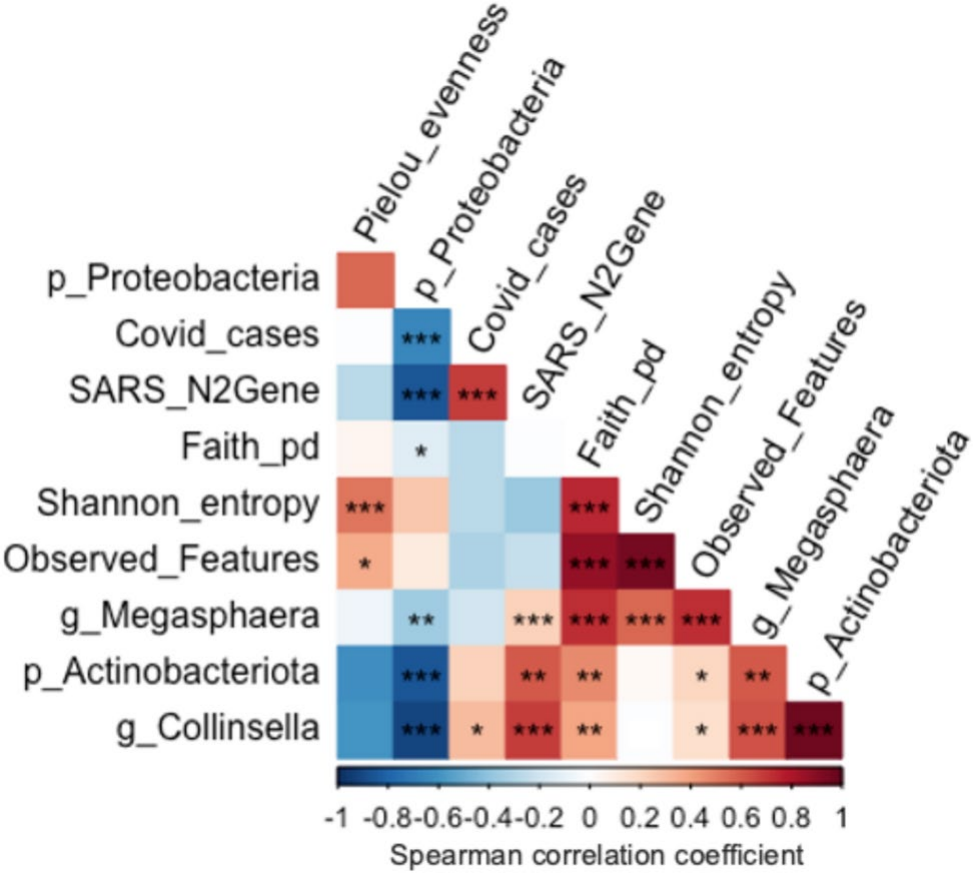
Spearman correlation heat matrix depicting associations between key microbial parameters (alpha diversities, relative abundance of four significant taxa), SARS-CoV-2 N2 gene wastewater concentrations, and 5-day rolling average of confirmed clinical COVID-19 cases across all samples throughout the entire study duration. Spearman correlation coefficient strength and direction are indicated with color scale. Significance levels indicated * < 0.05, ** < 0.01, and *** < 0.001

Across all samples, a summary depicting the associations between key microbial parameters (alpha diversity, relative abundance of the four significant taxa), SARS-CoV-2 N2 gene wastewater concentrations, and confirmed clinical COVID-19 5-day rolling averages is shown in the Spearman correlation heat matrix (Fig. 7). The relative abundance of genus *Megasphaera* positively correlates with three microbial alpha diversities (Faith’s Pd, Shannon’s Entropy, and Observed Features *p*-values ≤ 0.001). Two additional taxa, phylum Actinobacteriota and genus *Collinsella*, positively correlate with Faith’s Pd (*p*-values ≤ 0.01) and Observed Features (*p*-values ≤ 0.05). None of the four alpha diversities were shown to be significantly associated with SARS-CoV-2 wastewater concentrations or COVID-19 cases. Five-day rolling averages of confirmed clinical COVID-19 negatively correlate with phyla Proteobacteria (*p* ≤ 0.001) and positively correlate with genus *Collinsella* (*p* ≤ 0.05). These additional findings further confirm the strong associations between SARS-CoV-2 wastewater concentrations and the following four key taxa, Proteobacteria (–), Actinobacteriota (+), *Collinsella* (+), and *Megasphaera* (+) (*p*-values ≤ 0.01). An exploratory master table depicting Spearman correlation analyses between the relative abundance of the taxonomy of interest (*n* = 20) at the phylum and genus levels in association with quantitative (SARS-CoV-2 N2 gene concentrations (log GC/L), COVID-19 cases (5-day avg/100 k), microbial alpha diversity (Shannon’s Index), and wastewater human fecal indicators (PMMoV and crAssphage (log GC/L))) and qualitative parameters (WWTP and pandemic phase) can be found in Supplementary Table 3.

## Discussion

### Wastewater influent depicts core gut microbial communities of central Ohio cities

The majority of wastewater influent is composed of wastewater-associated microbiota (~ 88%), with approximately 12% originating from human-fecal gut-associated microbes (LaMartina et al. 2021). Therefore, in order to characterize the human gut bacterial communities via wastewater influent, we filtered taxa to retain only human gut-associated reads (defined as the bacterial taxa detected in the human intestinal tract) and omit wastewater-associated reads, which are known to undergo seasonal variation (Su et al. 2017). Our results show that a relatively high proportion (38%) of human gut-associated taxa were retained from urban wastewater influent, representative of more than 1.35 million Ohio individuals.

Across all 69 samples, the leading four phyla in descending order were Proteobacteria, Firmicutes, Actinobacteriota, and Bacteroidota, composing between 87.63 and 92.96% of the total microbial community (Supplementary Fig. 3A). Newark exhibited significantly higher relative abundances of Proteobacteria and Nitrospirota compared to Columbus (Supplementary Table 2). While Proteobacteria is one of the dominant gut-associated phyla and therefore contributes meaningfully to overall community structure, the difference between cities reflects a shift in relative proportion rather than a change in which phyla dominate the communities. In contrast, Nitrospirota represents a low-abundance phylum, indicating its higher relative abundance in Newark contributes minimally to overall phyla-level composition. Collectively, these findings show that although two of the phyla’s relative abundance differ statistically between cities, Columbus and Newark still share the same four dominant gut-associated phyla, indicating robustly similar phyla-level profiles across the populations in central Ohio served by Columbus and Newark WWTPs. This consistency aligns with prior wastewater microbiome studies across diverse geographic regions. Identical to our study, the same top four taxa, Proteobacteria, Firmicutes, Actinobacteriota, and Bacteroidota, were recognized as the four leading phyla in human bacterial communities from hospital sewage in Qatar (Johar et al. 2023). In a metagenomic study that compared the sewage microbiome of influent and effluent, Proteobacteria, Firmicutes, and Bacteroidota were synonymously identified as the top three phyla (Azli et al. 2022). Sewage influents across India and USA cities revealed that Proteobacteria, Bacteroidota, and Firmicutes roughly comprised between 81 and 96% of total microbial communities (Roguet et al. 2022; Singh et al. 2023). Across clinical gut microbiome studies, a similar pattern is accepted where these same four phyla predominate out of the more than 50 phyla that have been described (Rizzatti et al. 2017). Regardless of geographic variances, these studies support the conclusion that wastewater influent reliably captures the core gut microbiota at the phyla level.

In contrast, bacterial taxonomy diverged substantially at the genus level among WWTPs (Fig. 3) and city (Supplementary Fig. 3B). A total of 106 unique genera were retained across all samples following filtration against the Su et al. (2017) database of 382 unique genera. The top five contributing genera across all Jackson Pike, Southerly, and Newark WWTP influent samples were *Bifidobacterium*, *Leptotrichia*, *Subdoligranulum*, *Streptococcus*, and *Comamonas* (Supplementary Fig. 3A). However, these genera’s relative abundances vary drastically compared to literature. *Pseudomonas*, *Flavobacterium*, *Aeromonas*, *Tolumonas*, *Bacteroides*, and *Prevotella* consisted of the leading genera across wastewater influent in Johore, Malaysia, and as shown in Supplementary Fig. 3, these six genera were detected in all of this study’s 69 samples but were present in much lower relative abundances (Azli et al. 2022). *Bacteroides* is one of the most abundant taxa found in human stool but, interestingly, was not one of the highest representative genera in our study. Nonetheless, comparable *Bacteroides* relative abundances were detected around 3.4% in Milwaukee wastewater influent compared to an average relative abundance of 2.6% across all Jackson Pike, Southerly, and Newark WWTPs (LaMartina et al. 2021). Other genera, such as *Trichococcus* and *Acinetobacter*, were reported at relatively high abundances in Chinese urban sewage but were not detected in this study (Su et al. 2017). Subsequent differential abundance testing identified 10 genera enriched in Newark and seven genera enriched in Columbus (Supplementary Table 2). Unlike phyla, these findings indicate that genus-level profiles do not reflect a universal wastewater core. Microbial composition becomes increasingly community-specific at finer taxonomic resolution.

None of the four measures of alpha diversity (Shannon’s Entropy, Pielou’s Evenness, Faith’s Phylogenetic Diversity, and Observed Features Count) differed significantly between the city of Columbus and Newark (Wilcoxon rank sum *p*-value > 0.05). However, all four indices were relatively higher for the metropolitan Columbus city with population greater than 1.3 million compared to Newark city of 50,000 individuals. These Shannon entropy values (3.5–5.75) are comparable to the ranges reported from India sewage (3.6–6.67), in which urban regions showed higher alpha diversity than suburban or rural areas (Singh et al. 2023). Although alpha diversity was not drastically different, community dissimilarity significantly differed between Columbus and Newark cities, indicating distinct bacterial community structure by catchment (weighted UniFrac, PERMANOVA, *p* < 0.001). Collectively, while these findings show that phylum-level microbiota remain consistent across two central Ohio cities, finer taxonomic patters at the genus-level reveal more community-specific microbial footprints.

### Bacterial diversity: community structural changes during COVID-19 pandemic

COVID-19 cases and SARS-CoV-2 wastewater concentrations fluctuated throughout the 23-month study period without a consistent pattern (Fig. 1C, D). As expected, the two larger metropolitan WWTPs (Jackson Pike and Southerly) generally exhibited consistently higher case counts and viral loads compared to Newark WWTP. Three sharp increases in COVID-19 cases and two spikes in viral concentrations were detected during the months of December 2020/January 2021, September 2021, and December 2021/January 2022. We suspect that the first and greatest peak in cases and viral loads is attributed to the first wave of the Alpha variant B.1.1.7, which emerged in the USA in December 2020 (CDC, 2023). During the late summer of 2021, the Delta variant B.1.617.2 became the dominant variant in the USA, which may explain the second wave in COVID-19 cases. The third wave in COVID-19 cases and the second-largest peak in viral loads are likely attributed to the Omicron variant BA.1 (1.6 times more transmissible than the Delta variant) that began circulating in the USA in December 2021 (CDC, 2023). The temporal patterns in case counts and viral wastewater concentrations can be further contextualized by vaccination rates and circulating variants (Supplementary Fig. 1).

Despite fluctuations in COVID-19 burden (e.g., cases and viral loads), alpha diversity (Shannon’s Index) within WWTPs also varied longitudinally from August 2020 through June 2022 (Fig. 4A). Shannon’s Index differed significantly by pandemic phase (Kruskal–Wallis, *p*-value < 0.0001), with the pandemic phase having a higher alpha diversity index compared to both the transition and endemic phases (Wilcoxon, *p*-values < 0.001) (Fig. 4B). Likewise, Bray–Curtis distance matrices reveal significant clustering among pandemic phases (PERMANOVA *p*-value < 0.001, Fig. 4C). We hypothesize that these shifts in community structure may partially reflect the rapid introduction of COVID-19 vaccinations (Supplementary Fig. 1). Although COVID‑19 vaccines target viral infection, emerging evidence suggests immune-microbiome crosstalk as vaccinations can indirectly influence the gastrointestinal tract through immune modulation, systemic inflammatory signaling, and transient changes in host metabolic and physiological pathways (Lunken et al. 2024; Massad et al. 2025; Ng et al. 2022; Qiu et al. 2024). While very few clinical studies investigated the impact of SARS-CoV-2 vaccination on human gut microbiota, reports show short‑term reduction in gut microbial diversity and composition as soon as > 1 month following both mRNA (BNT162b2) and inactivated (CoronaVac) COVID-19 vaccines, therefore supporting biologically plausible mechanisms for community-level microbial changes across vaccination-defined phases (Lunken et al. 2022; Ng et al. 2022).

In our study region, cumulative vaccinations in Franklin and Licking County increased most rapidly during February/March 2021, coinciding with the sharp decline in community-level alpha diversity in Columbus and Newark from March to April 2021. It is important to note that alpha diversity did not continue decreasing after the transition phase but, instead, slightly increased. Thus, we suspect that after the quick vaccination rollout began, individuals’ gut dysbiosis slowly began to recover as we see this slow increase in alpha diversity at a population level from the transition vaccination phase into the endemic final plateau phase. This microbial recovery may also be attributed to waning immunity from mRNA vaccines, as protective antibodies can start fading as soon as 3–6 months following vaccinations (Nguyen et al. 2025). However, it is also possible that the significant reduction in community alpha diversity from March to April 2021 could also be a result of the largest wave of COVID-19 infection from the Alpha variant. Bacterial diversity was significant reduced in patients with COVID-19 compared to healthy controls and those with the seasonal flu (Gu et al. 2020; Tao et al. 2020). Therefore, we hypothesize that the COVID-19 alpha variant wave in tandem with vaccination introduction may have synergistically contributed to the observed temporary decrease in community alpha diversity.

Most notably, cumulative vaccination rates were highest during the transition phase when COVID-19 cases, SARS-CoV-2 wastewater concentrations, and alpha diversity were all at their lowest levels. We have shown that both alpha and beta diversity fluctuate throughout the COVID-19 pandemic in central Ohio, alongside varying rates of infection rates, wastewater viral loads, and vaccination patterns.

### Significant shifts in clinically relevant microbiota throughout the COVID-19 pandemic

We report significant differences in the relative abundance of five phyla and 15 genera across the pandemic phase (Fig. 5). Firmicutes, Actinobacteriota, and Euryarchaeota taxa were enriched during the pandemic phase, while Proteobacteria increased in the transition phase, and Verrucomicrobiota were more abundant in the endemic phase. Similarly, clinical observations have reported significant compositional changes in the gut microbiome of COVID-19 patients compared to healthy controls (Farsi et al. 2022). While one review suggested that the pandemic was generally characterized by lower phylum Actinobacteria relative abundances and higher Bacteroidetes richness (Zhang et al. 2023), another study contradicts this report by observing a relative increase in Actinobacteria among COVID-19 patients (Farsi et al. 2022). Meanwhile, two studies report significant reductions in Actinobacteria and Firmicutes following SARS-CoV-2 vaccination (Lunken et al. 2022; Ng et al. 2022). These studies may support the increased enrichment of phyla Actinobacteria and Firmicutes we observed during the pandemic phase of our study. The introduction of vaccinations at the end of the pandemic phase may have contributed to the subsequent population-level reduction of these phyla we observed during the transition and endemic phases, highlighting their relative enrichment in the pandemic phase.

At the genus level, 9 of the 15 unique genera identified were relatively more abundant during the pandemic phase, including *Turicibacter*, *Methanobrevibacter*, *Paracoccus*, *Collinsella*, *Megasphaera*, *Actinomyces*, *Bacteroides*, *Microbacterium*, and *Streptococcus*. Three genera were enriched during the transition phase, *Aeromonas*, *Tolumonas*, and *Dechloromonas*, and two genera were more abundant during the endemic phase, *Akkermansia* and *Catenibacterium*. Some of these notable taxa are reported in clinical studies that explore the human GM and COVID-19 infection. For example, an extensive systematic review examined 22 studies encompassing a total of 1255 confirmed COVID-19 patients and determined that all studies reported a significant association between gut microbiota dysbiosis and COVID-19 infection and/or severity (Farsi et al. 2022). In Farsi et al. (2022), general conclusions were made regarding the listed taxa below, with an asterisk representing the differential taxa that also overlapped with the differentially abundant taxa found above in this study. The greatest bacterial composition alteration across the selective 1255 COVID-19 patients was a reduction in genera *Alistipes*, *Ruminococcus*, *Bifidobacterium, *Eubacterium*, *Fusicathenibacter*, *Roseburia*, *Faecalibacterium*, and *Blautia*, and an enrichment of *Bacteroides, *Eggerthella*, *Actinomyces, *Megasphaera, *Clostridium*, *Collinsella, *Streptococcus, and *Rotia* (Farsi et al. 2022)*.* This supporting evidence may elucidate the enrichment of genera *Collinsella*, *Megasphaera*, *Actinomyces*, *Bacteroides*, and *Streptococcus* during the pandemic phase of our study (Farsi et al. 2022; Gallardo-Escárate et al. 2021; Tao et al. 2020; Zuo et al. 2020a).

Several of these genera are known opportunistic pathogens. The genus *Streptococcus* is an indicator of opportunistic bacterial invasion in the gut, and this taxa’s relative abundance was enriched during the pandemic phase and increases alongside COVID-19 infection (Donati Zeppa et al. 2020; Gu et al. 2020; Tao et al. 2020; Zuo et al. 2020b). Similarly, the genus *Collinsella* is another opportunistic pathogen commonly found in the gut of infected COVID-19 patients that increases with case severity and infectivity status, denoting its significantly enriched presence during the pandemic phase of this study when COVID-19 case rates were highest (Farsi et al. 2022; Liu et al. 2021; Massip Copiz 2021). Known to cause bacteremia, *Actinomyces* is an additional opportunistic gut pathogen we found enriched during the pandemic phase that has also been reported to increase in COVID-19 patients (Zuo et al. 2020b). *Bacteroides* is generally accepted as one of the most critical commensal bacterial genera found in the gut whose relative abundance alterations play a significant role in human health and disease outcome (Crovesy et al. 2020; Juárez-Fernández et al. 2021). The reduction of *Bifidobacterium* found in the GM of COVID-19 patients may aid in explaining the significant increase in *Bifidobacterium* we found during the transition phase, when community COVID-19 cases were at their lowest (Gu et al. 2020; Wu et al. 2021). Interestingly, members of the *Akkermansia* genus, known to protect against inflammation, were enriched during the endemic phase, potentially reflecting higher community vaccination coverage and immune protection (Zhang et al. 2023).

### Notable taxa in the potential role of SARS-CoV-2 infection

From the selective bacterial taxa that are differentially abundant by pandemic phase, four key taxa were identified as prospective wastewater biomarkers of human disease. These taxa significantly correlated with SARS-CoV-2 wastewater concentrations but do not correlate with fecal wastewater indicators (Fig. 6). Specifically, SARS-CoV-2 levels were negatively correlated with members of phylum Proteobacteria (*p* < 0.001) and were positively correlated with members of phylum Actinobacteriota (*p* ≤ 0.01) and the genera *Collinsella* (*p* ≤ 0.001) and *Megasphaera* (*p* ≤ 0.001) (Fig. 7). The positive association between the relative abundance of phyla Actinobacteriota and viral wastewater concentrations is consistent with a clinical reports that found increased Actinobacteria abundances in COVID-19 patients (Farsi et al. 2022). In contrast, the negative correlation between of Proteobacteria with COVID-19 cases and SARS-CoV-2 wastewater concentrations is unexpected. Proteobacteria is comprised of many known human pathogens, and with increased abundances are often indicative of an imbalanced gut microbiota (Rizzatti et al. 2017; Shin et al. 2015). Although we were expecting a positive association with COVID-19 infection since some of Proteobacteria are hypothesized as a signature of disease risk, Proteobacteria is the largest and most diverse phylum within the bacterial domain; therefore, it is difficult to determine with confidence which members of this phyla may be driving this negative association (Rizzatti et al. 2017). However, the relative abundance of Proteobacteria negatively correlated with Faith’s Pd (alpha diversity), indicating that enrichment of the phylum may be associated with reduced community evenness and potential gut microbiota imbalances. The genus *Megasphaera* has previously been reported in severe COVID-19 cases and has shown negative associations with SARS-CoV-2 vaccine responses, supporting our observed positive correlation with SARS-CoV-2 levels (Cao et al. 2021; Lunken et al. 2022). Similarly, we report that *Collinsella*, an opportunistic pathogen commonly detected at high abundances in the gastrointestinal tract of COVID-19 patients, positively correlated with both COVID-19 cases and SARS-CoV-2 titers (Liu et al. 2021; Massip Copiz 2021). Clinical gut studies have shown that *Collinsella* relative abundance strongly correlates with production of the proinflammatory cytokine IL-17A and suspect that this genus may contribute to the increased gut permeability and disease severity in autoimmune disorders (Chen et al. 2016; Ciccia et al. 2016). Given the well-established role of IL-17A in airway inflammation and host defense mechanisms, particularly by recruiting neutrophils, activating T cells, and enhancing antibody production in airway inflammation responses against pathogens, these findings potentiate the possibility that enriched *Collinsella* abundances may be linked to gut dysbiosis and SARS-CoV-2 infection dynamics (Iwakura et al. 2008).

We are surprised to find no significant correlation between alpha diversity and SARS-CoV-2 concentrations, considering one study found a reduced bacterial diversity in positive SARS-CoV-2 samples of campus dormitories wastewater compared to SARS-CoV-2 negative samples (Li et al. 2024). Although the strength of association was not significant, the direction of association is negative between measures of alpha diversity with SARS-CoV-2 wastewater levels and COVID-19 cases (Fig. 7). In tandem with significant differences in Shannon’s Index by pandemic phase (Wilcoxon, *p*-values < 0.001, Fig. 4B), these results may indicate broad community gut dysbiosis throughout the pandemic. Collectively, these microbial associations complement a growing body of clinical research showing that disruptions of the GM may impact respiratory infection outcomes through the gut-lung axis. Imbalances in the gut can modulate systemic inflammation, alter integrity of the epithelial barrier, and shape cytokine responses that are relevant to COVID-19 pathogenesis. Although wastewater surveillance cannot establish causality, our findings represent the first population-level evidence of biologically relevant patterns consistent with gut-lung crosstalk during the pandemic. These community-level shifts in gut microbiota could therefore reflect broader respiratory disease dynamics.

Beyond characterizing microbial patterns during the pandemic, wastewater-derived GM data offer a novel opportunity to monitor population-level biological responses to infectious disease stressors. Given the critical role of the GM in immune regulation, community-scale shifts in gut-associated taxa may reflect broader patterns in inflammatory status, chronic disease burden, environmental stressors, and recovery trajectories. Rather than informing individual behavior recommendations (e.g., diet, exercise, and stress management), this approach provides a scalable, non-invasive tool to identify communities experiencing disproportionate health risks, support early detection of emerging trends, and complement pathogen surveillance within traditional public-health systems. This broader framework highlights the potential of wastewater microbiome monitoring to advance community health assessment and pandemic preparedness.

### Limitations and future direction

Although wastewater-based epidemiology (WBE) offers a foundational framework for community-level health monitoring, this approach has several limitations. First, WBE is subject to ecological fallacy and the modifiable areal unit problem (MAP) since the microbial and viral signatures are characterized based on aggregate measurements. Thus, when interpreting wastewater surveillance results, it is critical to note that findings cannot be assumed to reflect the health status of all individuals within the catchment area. Most importantly, as an exploratory study, our analyses aimed to investigate the relative trends and associations between wastewater-derived microbiota and SARS-CoV-2 infection levels. Thus, it is vital to recognize that results do not establish causality. While SARS-CoV-2 was the primary circulating pathogen during the study period, other infectious diseases, such as circulating gastrointestinal infections (GIs) or seasonal enteric pathogens, could contribute to community-level gut microbial variation. Yet, GI disease surveillance data were not available at the sewershed scale for this study, but future work should integrate such data to help distinguish COVID-19 specific microbial shifts from those associated with other concurrent infections.

While we did not have access to WWTP influent chemical parameters (e.g., pH, conductivity, and chlorine residuals), we could not directly assess their influence on microbial composition. However, the sewershed-associated microbes of influent wastewater communities across 71 US cities have shown to be highly consistent, with temperature being the primary environment driver distinguishing warm and cold ecotypes (Roguet et al. 2022). Since Columbus and Newark are geographically proximate (< 50-mile distance) and fall within the same temperature-defined cold ecotype, there are unlikely to be major differences in sewer-associated environmental microbes. In addition, chlorine residuals dissipate rapidly within infrastructure plumbing and are greatly neutralized before wastewater reaches the WWTP influent where sampling occurred (Al-Jasser 2007; Odimayomi et al. 2026). Thus, chlorine exposure within the sewer system is unlikely to have heavily influenced the human gut-associated microbes analyzed in this study. Future work integrating chemical and operational WWTP data would provide greater insight.

Since the statewide OEPA and ODH wastewater monitoring network was designed in response to COVID-19, our study design does not include samples prior to the pandemic. One Newark sample (05/02/2021) yielded a SARS-CoV-2 non-detect, which is consistent with statewide wastewater surveillance trends rather than a sampling or analytical issue. According to the Ohio Wastewater Monitoring Network Dashboard (https://data.ohio.gov/wps/portal/gov/data/view/ohio-wastewater-monitoring-data?visualize=true), viral loads in Newark were at their lowest levels during the similar period April–July 2021. PMMoV and crAssphage were consistently detected across all samples indicating stable fecal loading, further confirming successful sample processing. As denoted in many wastewater surveillance studies, we were unable to control for potential confounding factors (i.e., diet, season, and behavior) that may also contribute to community gut microbiota variation. At the phylum-level diet, associated shifts are broad and non-specific (e.g., higher Firmicutes/Bacteroidota ratios with high-fat Western-style diets or increased Bacteroidota with higher fiber intake), and cuisine-specific phylum signatures are not well-defined (Magne et al. 2020; Zhang 2022). As behavioral factors including smoking, food access, and stress have plausible mechanistic influences on gut microbiota, individual data were not available at the sewershed scale (Gui et al. 2021; Madison and Kiecolt-Glaser 2019; Zhang 2022).

However, it is notable that our beta diversity analyses showed that the pandemic phase explained more variance in microbial community structure compared to seasonal stratification, suggesting that COVID-19–related societal and epidemiological dynamics may have been stronger drivers of community-level GM shifts (Supplementary Fig. 5). Though future WBE studies should consider, we could not evaluate dietary and behavioral information because the data was not available at the sewershed geographic unit. In our previous study, Ai et al. (2021) observed minimal instances where weekly WWTP flow rates were significantly increased and found that normalization of SARS-CoV-2 titers to human fecal indicators (PMMoV and crAssphage) was not necessary at our study sites. Thus, we interpreted that our longitudinal wastewater data was reasonably robust to the variation in sewage flow (precipitation events) and human fecal strength. In addition, we acknowledge the relatively low sequencing depth of human gut-derived microbiota detected from our wastewater influent samples. This is expected given the complexity and diversity of environmental matrices often resulting in fewer reads per sample compared to human-derived samples. While sufficient for detecting broad wastewater trends and associations, the sequencing depth limited our ability to investigate low-abundant taxa or conduct species-level analyses. Therefore, we encourage future wastewater surveillance studies to utilize deeper sequencing techniques to more accurately depict species-level microbiota relevant to human health. Lastly, our COVID-19 case data may reflect a potential source of bias. Studies have reported that official case counts in the USA likely underestimate SARS-CoV-2 infection rates (Bendavid et al. 2021; Havers et al. 2020). Underreporting may occur due to a variety of factors such as variations in testing methods, laboratory capacity, socioeconomic barriers to healthcare, and individuals with mild symptoms who recover on their own without seeking medical care (Reese et al. 2021). Nevertheless, coupling WBE with clinical case data can provide invaluable insights into population-level disease dynamics.

Despite variability across existing WBE microbiome studies, improving reproducibility and standardization remains a key priority. Future large-scale studies should adopt validated protocols for wastewater sampling, storage, processing, and sequencing to enhance comparability of high-quality samples across study locations and time points. In tandem with standardization procedures, we encourage utilizing deep sequencing methods (e.g., shotgun metagenomics) combined with multi-omics techniques (e.g., metabolomics and transcriptomics) to provide further insights into microbial function and its relation to disease burden. In doing so, these future advancements may enable the development of community-level microbiome health indices and for early detection of infectious disease outbreaks, antimicrobial resistance, or other emerging threats. The integration of novel applications, such as machine learning frameworks, may promote more improved prediction of disease patterns and health outcomes. Meanwhile, the “sociobiome” is an emerging concept in microbiome science that explores how a community’s microbiota composition of a particular geographic area is influenced by exposure to shared socioeconomic and environmental determinants (i.e., income, education, race/ethnicity, and pollutant exposure) (Kwak et al. 2024; Nobre and Alpuim Costa 2022). It is critical for future researchers to incorporate the sociobiome determinants of health when studying the microbiome-related health disparities. Lastly, as wastewater surveillance expands its reach, ethical considerations must remain central of future frameworks. Recent work highlights the importance of developing standardized ethical guidelines to ensure that surveillance is conducted in a responsible and justifiable matter, particularly in vulnerable communities (Coffman et al. 2021). Assimilating wastewater-based epidemiology into public health systems could support targeted, region-specific interventions (e.g., nutrition programs, probiotic effectiveness, and sanitation improvements) to enhance individuals’ gut microbiota composition while promoting overall well-being and reducing disease risk.

## Conclusions

In this exploratory large-scale microbiome study, we demonstrate that wastewater influent provides a novel and scalable approach for characterizing the collective gut microbiome of urban populations. Catchment communities in central Ohio exhibited distinct genus-level bacterial signatures and longitudinal community gut health changes during the COVID-19 pandemic, with significant changes in bacterial structure and diversity. Although not the primary focus, geographically proximate Ohio cities showed differences in community-level gut microbiota. We identified several taxa, including, but not limited to, *Collinsella*, *Megasphaera*, and members of Actinobacteriota that may play an intrinsic role in SARS-CoV-2 infection dynamics. Distinguished by its 23-month duration across two cities, this study represents the first longitudinal assessment of community gut microbiota during the COVID-19 pandemic and provides strong proof-of-concept that wastewater-based epidemiology can reflect community gut health and disease burden over time. As the first long-term population-level assessment of community gut microbiota during a global health crisis, these findings highlight the potential of wastewater surveillance to complement pathogen monitoring, capture host-associated biological signals, and support future public-health preparedness. Future work is critically needed to validate, standardize, and expand wastewater microbiome surveillance as a powerful and scalable tool for monitoring population-level gut health worldwide.

## Supplementary Information

Below is the link to the electronic supplementary material.

ESM 1 (DOCX 984 KB)

## Data Availability

The data are available at the National Center for Biotechnology Information (NCBI) Sequence Read Archive (SRA) under the accession number PRJNA1405769.

## Declarations

### Mandatory if your study involves humans and/or animals

This is not applicable.

### Clinical trial number

This is not applicable.
