# Supplementary material for: Exploring community-level gut health amidst COVID-19 pandemic: a proposed application of longitudinal wastewater surveillance
Source: Environ Sci Pollut Res Int. 2026 Aug 1;33(25):12730–50. doi: 10.1007/s11356-026-38012-3 (PMC13476289; doi:10.1007/s11356-026-38012-3)
Supplement: Supplementary file 1 [file 11356_2026_38012_MOESM1_ESM.docx]

Supplementary Information

**Exploring community-level gut health amidst COVID-19 pandemic: A proposed application of longitudinal wastewater surveillance**

Emma Lancaster^1,2^, Yuehan Ai^3^, Jiyoung Lee^1,3,4^ *

^1^ Division of Environmental Health Sciences, College of Public Health, The Ohio State University, Columbus, Ohio, USA

^2^ Environmental Sciences Graduate Program, The Ohio State University, Columbus, Ohio, USA

^3^ Department of Food Science & Technology, The Ohio State University, Columbus, Ohio, USA

^4^ Infectious Diseases Institute, The Ohio State University, Columbus, Ohio, USA

*Corresponding author: Jiyoung Lee, lee.3598@osu.edu

**Supplementary Table 1.** Summary of sample and microbiome information including library size and proportion of human gut-associated reads.

| Sample ID | WWTP | Date | Library size: All reads | Library size: Human gut reads | Human gut reads proportion |
| --- | --- | --- | --- | --- | --- |
| E01 | Jackson Pike | 8/9/20 | 30772 | 12588 | 0.41 |
| E02 | Southerly | 8/9/20 | 18767 | 8523 | 0.45 |
| E03 | Newark | 8/9/20 | 27500 | 10379 | 0.38 |
| E04 | Jackson Pike | 9/8/20 | 25581 | 7916 | 0.31 |
| E05 | Southerly | 9/8/20 | 26445 | 8824 | 0.33 |
| E06 | Newark | 9/8/20 | 17035 | 7120 | 0.42 |
| E07 | Jackson Pike | 10/4/20 | 17678 | 8625 | 0.49 |
| E08 | Southerly | 10/4/20 | 19533 | 8720 | 0.45 |
| E09 | Newark | 10/4/20 | 17719 | 9535 | 0.54 |
| E10 | Jackson Pike | 11/8/20 | 24560 | 4499 | 0.18 |
| E11 | Southerly | 11/8/20 | 21328 | 8619 | 0.40 |
| E12 | Newark | 11/8/20 | 12028 | 5221 | 0.43 |
| E13 | Jackson Pike | 12/1/20 | 19649 | 6445 | 0.33 |
| E14 | Southerly | 12/1/20 | 18553 | 5729 | 0.31 |
| E15 | Newark | 12/1/20 | 18339 | 8402 | 0.46 |
| E16 | Jackson Pike | 1/3/21 | 23645 | 9216 | 0.39 |
| E17 | Southerly | 1/3/21 | 18475 | 7124 | 0.39 |
| E18 | Newark | 1/3/21 | 16306 | 6714 | 0.41 |
| E19 | Jackson Pike | 2/2/21 | 21360 | 8506 | 0.40 |
| E20 | Southerly | 2/2/21 | 11920 | 4385 | 0.37 |
| E21 | Newark | 2/2/21 | 18746 | 7009 | 0.37 |
| E22 | Jackson Pike | 3/2/21 | 19326 | 6981 | 0.36 |
| E23 | Southerly | 3/2/21 | 23512 | 8769 | 0.37 |
| E24 | Newark | 3/2/21 | 14501 | 4854 | 0.33 |
| E25 | Jackson Pike | 4/4/21 | 9366 | 4027 | 0.43 |
| E26 | Southerly | 4/4/21 | 10308 | 4425 | 0.43 |
| E27 | Newark | 4/4/21 | 10148 | 3553 | 0.35 |
| E28 | Jackson Pike | 5/2/21 | 9141 | 4259 | 0.47 |
| E29 | Southerly | 5/2/21 | 13014 | 4456 | 0.34 |
| E30 | Newark | 5/2/21 | 12843 | 5089 | 0.40 |
| E31 | Jackson Pike | 6/1/21 | 13735 | 4664 | 0.34 |
| E32 | Southerly | 6/1/21 | 13597 | 5278 | 0.39 |
| E33 | Newark | 6/1/21 | 9495 | 4873 | 0.51 |
| E34 | Jackson Pike | 7/6/21 | 11890 | 3532 | 0.30 |
| E35 | Southerly | 7/6/21 | 15706 | 4258 | 0.27 |
| E36 | Newark | 7/6/21 | 9085 | 4380 | 0.48 |
| E37 | Jackson Pike | 8/1/21 | 16724 | 5065 | 0.30 |
| E38 | Southerly | 8/1/21 | 16238 | 5093 | 0.31 |
| E39 | Newark | 8/1/21 | 14707 | 5980 | 0.41 |
| E40 | Jackson Pike | 9/1/21 | 19896 | 6594 | 0.33 |
| E41 | Southerly | 9/1/21 | 8406 | 3524 | 0.42 |
| E42 | Newark | 9/1/21 | 7433 | 4111 | 0.55 |
| E43 | Jackson Pike | 10/3/21 | 7896 | 2540 | 0.32 |
| E44 | Southerly | 10/3/21 | 7196 | 2181 | 0.30 |
| E45 | Newark | 10/3/21 | 16097 | 6119 | 0.38 |
| E46 | Jackson Pike | 11/2/21 | 12597 | 3750 | 0.30 |
| E47 | Southerly | 11/2/21 | 13063 | 3858 | 0.30 |
| E48 | Newark | 11/2/21 | 16532 | 7526 | 0.46 |
| E49 | Jackson Pike | 12/7/21 | 21980 | 7224 | 0.33 |
| E50 | Southerly | 12/7/21 | 21019 | 8265 | 0.39 |
| E51 | Newark | 12/7/21 | 14232 | 4605 | 0.32 |
| E52 | Jackson Pike | 1/3/22 | 21273 | 10156 | 0.48 |
| E53 | Southerly | 1/3/22 | 24227 | 9372 | 0.39 |
| E54 | Newark | 1/3/22 | 12393 | 4463 | 0.36 |
| E55 | Jackson Pike | 2/1/22 | 13398 | 4852 | 0.36 |
| E56 | Southerly | 2/1/22 | 13480 | 4569 | 0.34 |
| E57 | Newark | 2/1/22 | 15799 | 3959 | 0.25 |
| E58 | Jackson Pike | 3/1/22 | 13773 | 4859 | 0.35 |
| E59 | Southerly | 3/1/22 | 19515 | 6569 | 0.34 |
| E60 | Newark | 3/1/22 | 10208 | 2908 | 0.28 |
| E61 | Jackson Pike | 4/3/22 | 14962 | 5412 | 0.36 |
| E62 | Southerly | 4/3/22 | 10429 | 4749 | 0.46 |
| E63 | Newark | 4/3/22 | 15050 | 6219 | 0.41 |
| E64 | Jackson Pike | 5/1/22 | 11039 | 5479 | 0.50 |
| E65 | Southerly | 5/1/22 | 20240 | 9206 | 0.45 |
| E66 | Newark | 5/1/22 | 13313 | 7001 | 0.53 |
| E67 | Jackson Pike | 6/1/22 | 14622 | 6105 | 0.42 |
| E68 | Southerly | 6/1/22 | 15208 | 5853 | 0.38 |
| E69 | Newark | 6/1/22 | 21472 | 10246 | 0.48 |
| AVG |  |  | 16,174.25 | 6,172.59 | 0.38 |

**Supplementary Table 2.** Differential abundance results of phyla and genera taxa by city and WWP in descending rank calculated using LefSe and ALDeX2.

|  | Phyla | | | | Genus | | | |
| --- | --- | --- | --- | --- | --- | --- | --- | --- |
| Difference by City | Columbus | | Newark | | Columbus | | Newark | |
|  |  | | Nitrospirota  Proteobacteria | | *Prevotella*  *Bifidobacterium*  *Lactococcus*  *Streptococcus*  *Faecalibacterium*  *Rhodococcus*  *Catenibacterium* | | *Enterococcus*  *Comamonas*  *Pseudomonas*  *Thauera*  *Paracoccus*  *Lactobacillus*  *Nitrospira*  *Vibrio*  *Nitrosomonas*  *Aerococcus* | |
| Difference by WWTP | Jackson Pike | Southerly | | Newark | Jackson Pike | Southerly | | Newark |
|  |  |  | | Nitrospirota | *Ruminococcus* | *Subdoligranulum*  *Leptotrichia*  *Veillonella* | | *Enterococcus*  *Comamonas Nitrospira*  *Paracoccus*  *Thauera*  *Lactobacillus*  *Actinomyces*  *Lactobacillus*  *Pseudomonas* |

**Supplementary Figure 1**. Timeline of wastewater influent sampling period (August 2020 – June 2022) with statewide government public health interventions, circulating SARS-CoV-2 variants, and proportion of Franklin (black line) and Licking (red line) county population with vaccinated individuals. Pandemic phases (pandemic, transition, endemic) are denoted at top.

**Supplementary Figure 2.** Correlation analyses between SARS-CoV-2 (gene copies/liter) N2 gene wastewater concentrations and clinical COVID-19 cases (5-day rolling average) within each WWTP location (Spearman correlation, p < 0.001).

A)

B)

**Supplementary Figure 3.** Relative abundance of bacterial taxa at A) phylum level by WWTP and, B) genus level by City.

A)

B)

***

**Supplementary Figure 4.** A) Alpha diversity (Shannon’s index) by city, not significant (NS). City average indicated via white diamonds. B) Beta diversity (Weighted UniFrac) by WWTP. PERMANOVA test value is shown via asterisks. Significance levels indicated by asterisks * < 0.05; ** < 0.01; *** < 0.001; **** < 0.0001.

A)

*

B)

***

Supplementary Figure 5. Beta diversity Weighted UniFrac by (A) season and (B) pandemic phase. PERMANOVA test value is shown via asterisks. Significance levels indicated * < 0.05; ** < 0.01; *** < 0.001.

**Supplementary Table 3.** Master biomarker correlation analyses between relative abundance of taxonomy of interest at phylum and genus level in association with qualitative and quantitative parameters. Global Kruskal-Wallis tests conducted between biomarkers by WWTP and pandemic phase with significant p-values indicated via yellow highlight. Spearman correlation tests conducted between biomarkers and SARS-CoV-2 N2 gene wastewater levels (log gene copies/liter), COVID-19 cases (5-day rolling average/100k), alpha diversity (Shannon’s index), and human fecal indicators, PMMoV and crAssphage (log gene copies/liter). Significance p-value < 0.05 was used with red highlight indicating significant negative (-) coefficient correlation, and green highlight indicating significant positive (+) coefficient correlation.

| Phylum | WWTP | Pandemic phase |  | SARS-CoV-2 N2 gene  (log GC/L) | | COVID-19 cases  (5-day avg/100k) | | Alpha diversity (Shannon’s Index) | | PMMoV  (log GC/L) | crAssphage (log GC/L) |
| --- | --- | --- | --- | --- | --- | --- | --- | --- | --- | --- | --- |
|  | Global Kruskal-Wallis | |  | Spearman Correlation | | | | | | | |
|  | *p* | *p* |  | *p* | Coefficient | *p* | Coefficient | *p* | Coefficient | Y/N correlation | |
| Actinobacteriota* | 0.170 | 0.005 |  | 0.010 | 0.310 | 0.090 | 0.210 | 0.087 | 0.210 | N | N |
| Firmicutes | 0.610 | 9.20E-07 |  | 0.015 | 0.330 | 0.190 | 0.160 | 0.009 | 0.320 | N | Y |
| Bacteroidota | 0.160 | 0.083 |  | 0.334 | -0.120 | 0.460 | 0.920 | 0.021 | 0.280 | N | N |
| ratio_Firmicutes_  Bacteroidota | 0.420 | 0.078 |  | 0.220 | 0.170 | 0.550 | 0.084 | 0.410 | 0.110 | N | N |
| Proteobacteria* | 0.011 | 0.001 |  | 0.001 | -0.400 | 0.002 | -0.380 | 0.048 | -0.270 | N | N |
| Verrucomicrobiota | 0.880 | 2.20E-09 |  | 0.300 | 0.130 | 0.860 | -0.023 | 0.004 | -0.350 | Y | N |
|  | | | | | | | | | | | |
| Genus | | | | | | | | | | | |
| *Actinomyces* | 0.020 | 0.006 |  | 0.990 | -0.002 | 0.244 | -0.144 | 0.190 | 0.160 | N | N |
| *Bacteroides* | 0.250 | 0.022 |  | 0.368 | -0.111 | 0.589 | 0.067 | 0.083 | 0.210 | N | N |
| *Bifidobacterium* | 0.083 | 0.041 |  | 0.016 | 0.290 | 0.051 | 0.240 | 0.640 | 0.057 | N | Y |
| *Collinsella** | 0.580 | 5.10E-05 |  | 0.002 | 0.370 | 0.020 | 0.279 | 0.031 | 0.260 | N | N |
| *Enterococcus* | 2.70E-10 | 0.160 |  | 0.650 | 0.060 | 6.70E-07 | -0.560 | 0.810 | 0.030 | Y | N |
| *Faecalibacterium* | 0.002 | 0.058 |  | 0.145 | 0.178 | 0.010 | 0.312 | 0.006 | 0.330 | N | Y |
| *Lactobacillus* | 0.002 | 0.014 |  | 0.140 | 0.183 | 0.039 | -0.250 | 0.588 | 0.066 | N | N |
| *Megasphaera** | 0.860 | 4.60E-07 |  | 0.001 | 0.400 | 0.400 | 0.110 | 2.50E-04 | 0.430 | N | N |
| *Parabacteroides* | 0.031 | 0.500 |  | 0.910 | -0.012 | 0.590 | 0.067 | 0.005 | 0.335 | N | N |
| *Prevotella* | 0.004 | 0.500 |  | 0.970 | 0.004 | 0.036 | 0.260 | 0.002 | 0.360 | Y | N |
| *Ruminococcus* | 0.004 | 0.740 |  | 0.500 | 0.084 | 0.700 | 0.049 | 0.002 | 0.370 | N | N |
| *Streptococcus* | 0.089 | 0.022 |  | 0.380 | 0.110 | 0.011 | 0.310 | 0.005 | 0.330 | N | N |

* key biomarker that fulfills criteria: correlates 1) pandemic phase and 2) SARS-CoV-2 levels, and 3) does not correlate with fecal indicators.
